# Through Predictive Personalized Medicine

**DOI:** 10.3390/brainsci10090594

**Published:** 2020-08-28

**Authors:** Giuseppe Giglia, Giuditta Gambino, Pierangelo Sardo

**Affiliations:** 1 Department of Biomedicine, Neuroscience and Advanced Diagnostics (BiND), Section of Human Physiology, University of Palermo, 90134 Palermo, Italy; giuseppe.giglia@unipa.it (G.G.); pierangelo.sardo@unipa.it (P.S.); 2 Euro Mediterranean Institute of Science and Technology, I.E.ME.S.T, 90139 Palermo, Italy

**Keywords:** neuroblastoma, PD-L1, computational modelling, immunotherapy

## Abstract

Neuroblastoma (NBM) is a deadly form of solid tumor mostly observed in the pediatric age. Although survival rates largely differ depending on host factors and tumor-related features, treatment for clinically aggressive forms of NBM remains challenging. Scientific advances are paving the way to improved and safer therapeutic protocols, and immunotherapy is quickly rising as a promising treatment that is potentially safer and complementary to traditionally adopted surgical procedures, chemotherapy and radiotherapy. Improving therapeutic outcomes requires new approaches to be explored and validated. In-silico predictive models based on analysis of a plethora of data have been proposed by Lombardo et al. as an innovative tool for more efficacious immunotherapy against NBM. In particular, knowledge gained on intracellular signaling pathways linked to the development of NBM was used to predict how the different phenotypes could be modulated to respond to anti-programmed cell death-ligand-1 (PD-L1)/programmed cell death-1 (PD-1) immunotherapy. Prediction or forecasting are important targets of artificial intelligence and machine learning. Hopefully, similar systems could provide a reliable opportunity for a more targeted approach in the near future.

Amongst the deadly diseases affecting children, neuroblastoma (NBM) accounts for 15% of all deaths in pediatric age [1]. It is the most common tumor of the sympathetic nervous system, often evidenced as an extracranial mass that is most frequently encountered in the adrenal glands [2]. NBM cell lines are frequently used for basic research that investigates synaptic function and the relative pharmacological modulation [3,4]. This approach has been widely applied in literature to explore fundamental processes of the nervous system and neurodegenerative trajectories [5,6]. It is estimated that 6% of pediatric cancers are due to NBM, with a mean age of diagnosis of 1–2 years old and rare over the age of 10 [7,8]. However, the behavior of this tumor dramatically changes according to both host factors, such as age at diagnosis, and tumor biology, ranging from a high probability of spontaneous regression to 41.5% survival five years after diagnosis [9].

For this reason, a huge effort has been made by many researchers and clinicians in order to perform a risk stratification of patients into groups with low, intermediate, and high risk; moreover, it has been recently proposed to add a ‘ultra-high risk’ group that causes “death from disease within 18 months of diagnosis” [10].

According to the latest data, about 50% of patients affected by NBM are classified as a high-risk group [11]. As a consequence, early identification of high or ultra-high-risk groups should lead to more aggressive treatments, whereas the goal of recognizing low-risk groups is to avoid unnecessary treatments and ultimately minimize adverse effects [12]. Among biological factors related to higher risk, in 2009 the International Neuroblastoma Risk Group (INRG) mentioned tumor histology and its grade of differentiation, DNA index (ploidy) and MYCN oncogene amplification (MYCN-A) [13].

More recently, a better understanding of the biology and pathophysiology of NBM has been reached thanks to the identification of intracellular molecular cascades and signaling pathways. These have been associated with the control of cell growth and division, mutations, phenotypic changes and ultimately responsible for the development of cancer [14]. The identification of immune checkpoints, playing a crucial role in the transformative potential of tumors, has quickly boosted the development of immunotherapy approaches to different cancer types, for instance acting on excessive damage to tissues induced by inflammation [15].

It has been speculated that programmed cell death-protein-1 (PD-1) can modulate immune response by binding to programmed cell death-ligand-1 (PD-L1), a transmembrane protein expressed in T cells. On this point, natural killer cells (NK) are promising immune effectors in anticancer responses mediated by T cells [11]. This immune checkpoint, with associated signaling pathways, has been identified as a target for anticancer drugs for monoclonal antibodies, nivolumab and pembrolizumab, and small molecules [16]. When combined with anti-GD2 antibodies, nivolumab has been proven to be effective in a mouse model of NBM [17]. Further research described response rates to the PD-1/PD-L1 axis blockade, suggesting that PD-L1 expression may also serve as a predictive marker in small-cell lung cancer and NBM [18,19].

Altogether, these findings point to the possibility of combining the computing power and clinical data to realize predictions of the response to immunotherapy treatments. In order to identify and assess the impact of specific parameters and targets, predictive models provide solid bases for developing well-directed and targeted approaches. Specifically, in the paper by Lombardo et al. [20], Michelis–Menten modified equations were employed to evaluate the parameters that mostly affect the expression of PD-L1 by means of computer-based sensitivity analyses. The results of this study revealed the different influence of ALK- and EGFR-pathways in the expression of PDL-1, depending on the genetic targets implicated. For instance, this kind of prediction indicates that patients with ALK1174L-mutated tumors would better respond to a therapy with nivolumab rather than crizotinib. 

It is worth noting that in a very recent study two NBM patients were treated with a combination of nivolumab and anti-disialoganglioside antibody dinutuximab beta (DB), which is known to exert its function by modulating PDL-1 expression. Both patients had a remarkable improvement of clinical picture [21]. Accordingly, the most up-to-date studies on both nivolumab and pembrolizumab proved their safety as an anticancer treatment for pediatric patients. Nonetheless, despite their promising preclinical profile, they showed notable effects only on lymphoma and not on NBM patients. In detail, nivolumab was used in solid tumors without pre-screening for PDL-1 and only 7% of NBM (one out of 15 patients) was PDL-1 positive [22]. Pembrolizumab was instead used in young patients who were tumor PDL-1 positive, but the response rate was similar to that obtained in the non PDL-1 screened population, thus leading the authors to conclude “PD-L1 expression alone is not sufficient as a biomarker for the selection of paediatric patients who are likely to respond to PD-1 checkpoint inhibitors” [23].

Taken together, the aforementioned papers give strength to the Lombardo model [20], which could explain these apparently contrasting findings. According to this model, the ALK gene is the main determinant of PDL-1 expression. Hence, in patients who are both PDL-1 positive and a carrier of ALK1174L mutation, nivolumab results in being more effective with respect to patients that do not carry this mutation. 

We believe that this new approach, based on in-silico simulation, has great potential especially in those illnesses in which precision medicine has a primary role, like NBM [12].

Moreover, this network model of molecular pathways could generate a sufficient bulk of simulated data of interaction, when real ones are unavailable. This can be put into practice in order to significantly increase the reliability of decision-making algorithms based on machine learning (ML), which strongly depends on the amount of datasets involved. This would begin a virtuous cycle, allowing faster experimentation and targeted treatment. Indeed, a recent work by Schmauch et al. [24] showed that the He2RNA model can be trained to correctly predict RNA sequences profile from whole-slide images of different types of tumor. Exploiting the power of ML and computational models of molecular pathways would set the basis for a future in computer-aided precision medicine.
